# A tailored intervention to implement guideline recommendations for elderly patients with depression in primary care: a pragmatic cluster randomised trial

**DOI:** 10.1186/s13012-016-0397-3

**Published:** 2016-03-09

**Authors:** Eivind Aakhus, Ingeborg Granlund, Jan Odgaard-Jensen, Andrew D. Oxman, Signe A. Flottorp

**Affiliations:** 1 Research Centre for Old Age Psychiatry, Innlandet Hospital Trust, N-2312 Ottestad, Norway; 2 Norwegian Knowledge Centre for the Health Services, Box 7004, St Olavs plass, N-0130 Oslo, Norway; 3 The Department of Health Management and Health Economics, University of Oslo, P.O. box 1130, Blindern, 0318 Oslo, Norway

**Keywords:** Depression, Elderly, Primary care, Tailored implementation interventions, Implementation science, Cluster randomised trial

## Abstract

**Background:**

Elderly patients with depression are underdiagnosed, undertreated and run a high risk of a chronic course. General practitioners adhere to clinical practice guidelines to a limited degree. In the international research project Tailored Implementation for Chronic Diseases, we tested the effectiveness of tailored interventions to improve care for patients with chronic diseases. In Norway, we examined this approach to improve adherence to six guideline recommendations for elderly patients with depression targeting healthcare professionals, patients and administrators.

**Methods:**

We conducted a cluster randomised trial in 80 Norwegian municipalities. We identified determinants of practice for six recommendations and subsequently tailored interventions to address these determinants. The interventions targeted healthcare professionals, administrators and patients and consisted of outreach visits, a website presenting the recommendations and the underlying evidence, tools to manage depression in the elderly and other web-based resources, including a continuous medical education course for general practitioners. The primary outcome was mean adherence to the recommendations. Secondary outcomes were improvement in depression symptoms as measured by patients and general practitioners. We offered outreach visits to all general practitioners and practice staff in the intervention municipalities. We used electronic software that extracted eligible patients from the general practitioners’ lists. We collected data by interviewing general practitioners or sending them a questionnaire about their practice for four patients on their list and by sending a questionnaire to the patients.

**Results:**

One hundred twenty-four of the 900 general practitioners (14 %) participated in the data collection, 51 in the intervention group and 73 in the control group. We interviewed 77 general practitioners, 47 general practitioners completed the questionnaire, and 134 patients responded to the questionnaire. Amongst the general practitioners who provided data, adherence to the recommendations was 1.6 percentage points higher in the intervention group than in the control group (95 % CI −6 to 9).

**Conclusions:**

The effectiveness of our tailored intervention to implement recommendations for elderly patients with depression in primary care is uncertain, due to the low response rate in the data collection. However, it is unlikely that the effect was large. It remains uncertain how best to improve adherence to evidence-based recommendations and thereby improve the quality of care for these patients.

**Trial registration:**

ClinicalTrials.gov: NCT01913236.

**Electronic supplementary material:**

The online version of this article (doi:10.1186/s13012-016-0397-3) contains supplementary material, which is available to authorized users.

## Background

Depression in the elderly is common, and the prevalence increases with age [1, 2]. Once suffering from a depressive episode, the prognosis is poor for 20–50 % of the patients [3]. In a 3-year follow-up study, Denihan and colleagues found that more than half of depressed elderly patients had persistent depressive symptoms or sub-threshold symptoms [4]. Collaborative care alleviates depressive symptoms and anxiety in the elderly, as well as other patients [5, 6]. Other multifaceted interventions also effectively improve depression, at least in the short term [7].

Clinical practice guidelines for the management of depression are available for primary and specialist healthcare in many countries. Adherence to guidelines improves outcomes for depressed patients [8]. Still, general practitioners adhere to clinical practice guidelines to a limited degree only. Smolders and colleagues [9] found that only 42 % of depressed patients received treatment in accordance with a Dutch clinical practice guideline for depression management. Subjectively, perceived adherence amongst general practitioners is considerably higher [10]. Although the Norwegian clinical practice guideline for depression treatment in adults recommends that second generation antidepressants should be used as the drug of choice when initiating antidepressant therapy, general practitioners prescribe first-generation antidepressants to a larger extent for their older patients, in particular for 60- to 79-year-olds [11].

Tailoring implementation strategies to identified determinants of practice is more effective than passive dissemination of guidelines or no intervention at all [12]. Still, there is uncertainty regarding how best to tailor the interventions. In the Tailored Implementation for Chronic Diseases (TICD) collaborative research project, we have developed and compared alternative methods for identifying determinants of practice and linking implementation strategies to identified determinants across countries and chronic diseases [13–16].

As there was no clinical practice guideline for managing depression in the elderly in Norway, only a national guideline on management of depression amongst adults in general [17], we conducted a systematic review, assessing 13 national and international clinical practice guidelines for managing depression in primary care [18]. We identified all relevant recommendations regarding elderly patients with depression. In collaboration with a reference group (see Acknowledgements and Table 1), we prioritised implementation of six of these in primary care in Norway. The objective of this study was to evaluate the effectiveness of tailored interventions to implement those six recommendations. Our main research question was as follows: does a tailored implementation strategy increase the extent to which general practitioners adhere to evidence-based recommendations for managing elderly patients with depression compared to no intervention?

**Table 1:** Six prioritised recommendations for the management of depression in elderly patients

Prioritised recommendations	Full recommendation to be discussed in the groups and interviews
1. Social contact	Primary care physicians and other healthcare professionals should discuss social contact with elderly patients with depression and recommend actions (e.g. group activities) for those who have limited social contact. •When needed, regular social contact with trained volunteers, recruited from Centres for Voluntary Organisations, the Red Cross, Mental Health or community day care centres. When possible, the patient’s relatives should be involved in the plan to improve social contact.
2. Collaborative care plan	All municipalities^a^ should develop a plan for collaborative care for patients with moderate to severe depression. The plan should describe the responsibilities and communication between professionals who have contact with the patient, within primary care and between primary and specialist care. In addition, the plan should appoint depression care managers who have a responsibility for following the patient. The plan should describe routines for referral to specialist care.
3. Depression care manager	Primary care physicians should offer patients with moderate to severe depression regular contact with a depression care manager.
4. Counselling	Primary care physicians or qualified healthcare professionals should offer advice to elderly patients with depression regarding: • Self-assisted programmes, such as literature or web-based programmes based on cognitive behavioural therapy principles • Structured physical activity programmes, individually or group-based • Healthy sleeping habits • Anxiety coping strategies • Problem solving therapy
5. Mild depression	Primary care physicians should usually not prescribe antidepressants to patients with mild depression. Primary care physicians may consider prescribing antidepressant medication to patients who suffer from a mild episode of depression and have previously responded to antidepressant medication when moderately or severely depressed.
6. Severe depression, recurrent depression and dysthymia	Primary care physicians should offer these patients a combination of antidepressant medication and psychotherapy. If the physician is not trained to provide the patient with psychotherapy, patients should be referred to trained healthcare professionals.

^a^Municipalities are the atomic unit of local government in Norway and are responsible for outpatient healthcare services, senior citizen services and other social services. There are 428 municipalities

## Methods

For a full description of our methods, please refer to the protocol [19]. In the following section, we briefly describe our methods and the deviations from the protocol.

### Design

We conducted a pragmatic cluster randomised trial comparing implementation of the six recommendations using tailored interventions with no intervention. The trial was pragmatic in that it attempted to answer a practical management question in normal practice, including all general practices in the targeted municipalities and all patients with the targeted condition, the intervention was delivered flexibly and the primary outcome measure was clinically meaningful [20, 21]. We randomised 80 out of the 428 municipalities in Norway into one of two groups: an intervention group, to which we delivered a tailored intervention to implement the six recommendations, and a control group, to which we did not deliver any intervention before the end of the data collection. We included 80 municipalities due to the required number of general practitioners that should be included in the study. The municipalities were selected from seven of 19 counties in Norway for practical reasons (geographical access by the research team) and because they represented both urban and non-urban and large and small municipalities.

### Participants, inclusion and eligibility criteria

At the patient level, we included home-dwelling elderly patients, 65 years or older, with a diagnosis of mild, moderate or severe depressive episode, recurrent depression or dysthymia according to standardised ICD 10 criteria [22], who had consulted their practitioner within the last 6 months before the intervention. At the healthcare professional level, we offered the intervention to all general practitioners in the intervention group, and we invited all general practitioners in the intervention and the control municipalities to participate in the data collection. We identified the general practitioners in the included municipalities using nationally available online registers, and we invited them by mail, email or phone. For general practitioners who consented to participate, we identified eligible patients from the general practitioners’ patient lists using software that extracted information from their electronic medical records, based on an algorithm of ICPC-2 diagnostic codes, ICPC-2 diagnostic text, free text, prescription of antidepressants and billing codes. We developed software for the five electronic journal systems used by general practitioners in Norway. The software was either available from the website and could be downloaded and installed by the practitioner prior to the interview or was sent on a memory stick beforehand. If the software did not work (e.g. installation of software was denied due to security systems, such as a firewall, or a patient list was not produced), we asked the general practitioners to think of elderly patients with depression from their list. Approximately one out of five general practitioners experienced some problems while installing the software. Nearly all of these general practitioners whom we interviewed were able, nonetheless, to identify elderly patients with depression from their practice. In the final stage of the data collection, we also asked general practitioners who had not responded to our invitation, to complete a questionnaire regarding their diagnosis and management of one elderly patient with depression from their patient list. General practitioners who participated in the interview received credit for the continuous medical education course but did not receive any financial compensation. We excluded patients if they did not have a diagnosis of depression according to ICD-10, if they had a diagnosis of dementia or bipolar disorder or resided in nursing homes or if their practitioner assessed them to have low life expectancy.

### Interventions

We developed a multicomponent intervention using a logic model to establish the hypothesised causal links between the components, the determinants, the recommendations and the expected improved adherence. We have provided details regarding how we developed the intervention in a study describing the tailoring process [23]. We grouped the components into six domains (Table 2).

**Table 2:** Strategies of the implementation programme

Strategy		Content
1	Collaborative care plan. Resources for administrators and policy makers: development of the plan	Help for healthcare administrators to develop a collaborative care plan in the community, presented on a designated website for the project.
2	Collaborative care plan. Resources for administrators and policy makers: content of the plan	Suggested content to include in the collaborative care plan, with an option to adjust or tailor interventions to the community.
3	Resources for healthcare professionals	Templates, manuals and pamphlets to be distributed to healthcare professionals in the municipality.
4	Resources for patients, their relatives and volunteers	Pamphlets to be distributed to patients and their relatives.
5	Outreach visits to general practitioners	Visits to general practitioners to provide information on the recommendations and determinants of practice and to discuss local considerations that might imply that the interventions should be adjusted or targeted to selected practices.
6	Web resources and data systems	A comprehensive website that includes the recommendations and the underlying evidence, tools for diagnosis and treatment and all educational resources, available for healthcare professionals and inhabitants in the intervention municipalities.

**Fig. 1:**
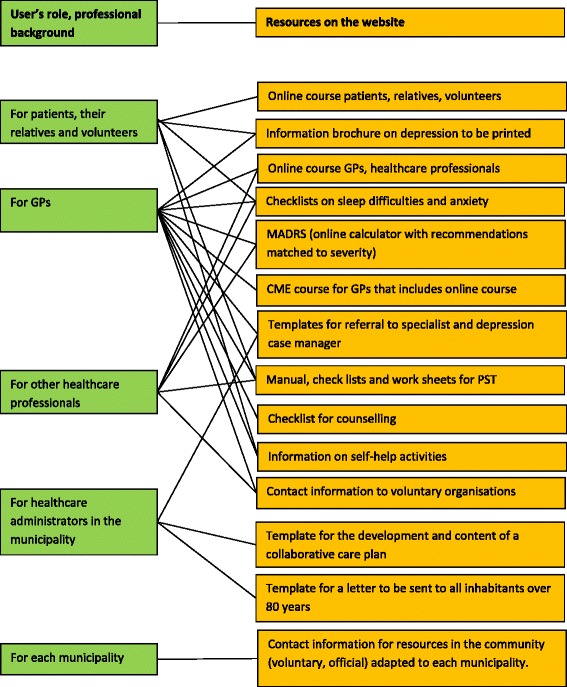
Content and structure of the website (www.depresjonhoseldre.no) [24]. The website has been subject to adaptations and adjustments after the intervention. It is in Norwegian

We delivered the intervention to the 40 municipalities in the intervention group. We conducted outreach visits to general practitioners. We developed a website that provided the recommendations, the underlying evidence for the recommendations, tools to diagnose and manage elderly patients with depression and online courses. We developed one online course for healthcare professionals (approved by the Norwegian Nurses Organisation and Norwegian Union of Municipal and General Employees for 8 h and by the Norwegian Medical Association as part of a continuous medical education (CME) course) and one for patients and their relatives (Fig. 1) [24]. Depending on which municipality the professionals or users worked or lived in, they received information adapted to that particular municipality in addition to general information that applied to all municipalities. Additionally, the online users received tailored information based on their profession or relation to the healthcare service (general practitioner, nurse, healthcare administrator, patient and their relatives). The website provided information for general practitioners on how to complete a CME course approved by the Norwegian Medical Association for 15 h. We also provided paper-based information that we distributed at outreach visits and by post to the municipalities.

### Outcome measures

The primary outcome was the proportion of the six recommendations adhered to by the general practitioners. We measured this outcome by calculating a single measurement for each general practitioner based on the mean adherence across the six recommendations.

Secondary outcomes were:

- Improvements in depression symptoms, as measured by the general practitioner, patient or family members. The general practitioners used the Clinical Global Impression improvement scale [25]. Patients’ assessment of continuous depression and anxiety symptoms was measured using the Hospital Anxiety and Depression Scale [26], and patients’ or family members’ assessment of improvements in depression symptoms was measured using the Patient’s Global Impression scale [27, 28].
- Patients’ feeling of loneliness [29, 30].
- Social contact.
- Physical activity, sleep problems management.
- Anxiety problems management.
- Problem solving ability.
- Utilisation of web- or book-based self-help programmes.
- Self-reported adherence to antidepressant therapy [31, 32].

In addition to what we described in the protocol [19], we included a calculation of adherence for additional depression subgroups (recurrent depression and dysthymia). On the municipality level, we recorded whether the municipality had developed a collaborative care plan for the adult population or for elderly patients with depression.

### Sample size

Our power calculations indicated that we would need to recruit 437 general practitioners in a sample of 80 municipalities, providing data for a maximum of 2622 patients (up to six patients per general practitioner), to detect a minimum difference in adherence to the recommendations amongst general practitioners of 5 %. This was based on an assumed standard deviation of 17 %, an intra-class correlation coefficient of 0.02, and 50 % of the general practitioners consenting to data collection. We based the assumed 50 % participation rate in data collection on two previous studies in Norwegian primary care [33, 34]. The detailed results of our power calculations are provided in appendix D in the protocol [19].

### Recruitment, randomisation and blinding

The statistician (JOJ), who was not otherwise involved in the study before the analysis, randomised the municipalities and assigned computer-generated random numbers to all 80 municipalities. We divided the municipalities into four strata based on two factors: (1) municipalities with city status or a large population (>25,000 inhabitants) versus ones with smaller populations (≤25,000 inhabitants) and (2) municipalities with a high proportion (>5 %) versus a low proportion (≤5 %) of inhabitants 80 years or older. There are few large municipalities in Norway. There is generally more access to cognitive behavioural therapy and other health and social services in large municipalities. Because we believed that there might be an association between access to health and social services and the primary outcome measure, we stratified the randomisation based on the size of the municipality to ensure that large municipalities were evenly distributed in the intervention and comparison arms of the trial. Furthermore, we stratified the randomisation on the variable “Proportion of inhabitants 80 years or older”, acknowledging the increased prevalence of depression amongst the oldest, which may influence the general practitioners’ experience with and skills of managing elderly patients with depression. The choice of the cut-off (≥25,000 inhabitants or designated city status) was based on data from Statistics Norway, reported in Appendix C of the protocol [19]. The characteristics of the included municipalities are shown in appendix C in the protocol.

Blinding of the participants and the researchers regarding the intervention was not possible. The statistician (JOJ) was blinded to the randomisation when he performed the analyses for the primary and secondary outcomes described in the protocol. We performed post hoc analyses after breaking the randomisation code.

### Data collection

We sought consent to collect data from all general practitioners in the 80 municipalities after we had delivered the interventions. We realised that the planned interview schedule (six patients per general practitioner) was too extensive. Thus, we reduced the number of patients that we wanted to discuss with each GP to four, aiming to conclude the interview within 1 h. The initial part of the interview served to decide whether the patients could be included and to assess depression severity and type and then to assess the practitioners’ management of the patient’s depression. We encouraged the general practitioners to provide diagnoses for two additional patients. Patients that we discussed with the general practitioner received information about the study and a questionnaire that we sent via their practitioner. Due to the poor response rate to the interviews with the general practitioners, we created a questionnaire and invited all general practitioners who had not previously responded in both groups to provide information for the management of one elderly patient with depression.

We did not collect baseline data on adherence to the recommendations, assuming that both groups were comparable following the stratification and randomisation process. From the general practitioner we collected the following descriptive data: age, gender, years of clinical experience as a general practitioner, whether the general practitioner was a specialist in general practice, the size of the general practitioner’s practice (number of patients on the general practitioner’s list), access to cognitive therapy in the community (easy/difficult), and the general practitioners’ subjective assessment of the proportion of elderly patients on their lists (many/few as compared with colleagues). We did not record the number of general practitioners with cognitive behavioural therapy skills or the exact number of elderly patients on the general practitioners’ lists, as described in the protocol.

We recruited and trained medical students who interviewed the general practitioners by phone. The general practitioners, but not the interviewer, had access to the patients’ medical records during the interview.

A time schedule for the data collection is presented in the protocol, appendix E [19].

### Statistical methods

We assessed the proportion of recommendations that the general practitioner adhered to, taking into account the severity and type of depression. For mild and moderate depression, there were seven items each to be fulfilled to qualify for adherence, and for severe depression, there were two items [19]. Although not described in the protocol, we included two items for recurrent depression and dysthymia.

We performed the analysis as an intention-to-treat analysis based on the available data; all general practitioners were analysed in the group to which they were assigned, regardless of whether they used the interventions we offered or not. We did not impute values for missing data. All analyses were performed using IBM SPSS v.21 with random effects for municipality and practice to account for the clustered nature of the data. We used linear regression for continuous data and logistic regression for dichotomous data. In the initial analyses, only the allocation to intervention or control and the two stratification variables (city/large population vs. small population and high vs. low proportion of inhabitants 80 years or older) were included as independent variables. We assumed that the following factors were potential effect modifiers: whether the municipality already had a collaborative care plan, whether access to cognitive behavioural therapy was poor, whether municipalities belonged to the “Centre for Development of Institutional and Home Care Services” [35] network and whether general practitioners had many elderly patients on their lists. We included each of the pre-specified effect modifiers separately as independent variables (alongside allocation to intervention or control group) in the model. All the effect modifiers with *p* < 0.3 in the previous step were included as independent variables in a final multivariate model.

### Ethics

General practitioners and patients who participated in data collection gave informed consent. The research group did not know the identity of the patients that we discussed with the general practitioner until the patients returned the questionnaire. The project was approved by the Regional Ethical Committee of the South-Eastern region of Norway (file n° 2013/572b).

## Results

**Fig. 2:**
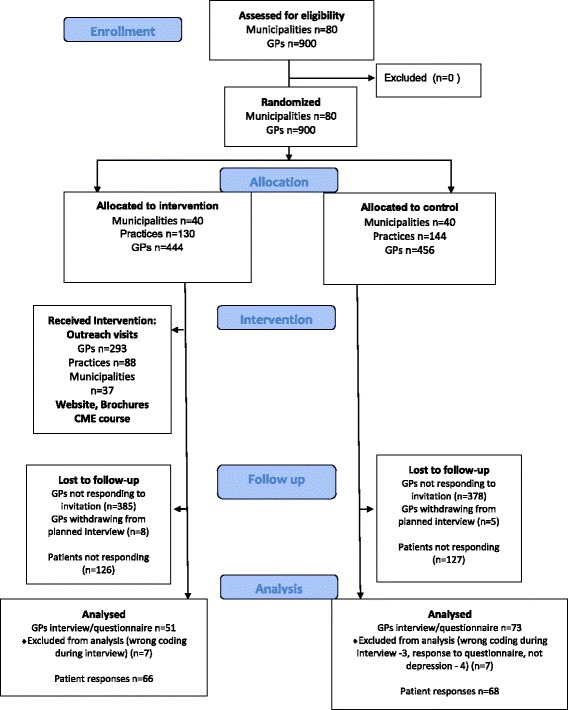
Consort 2010 flow chart of participants

Figure 2 is a flow chart showing the recruitment of general practitioners for the data collection to assess the effectiveness of the interventions. The intervention was from October 2013 to April 2014. Recruitment of participants and data collection was from May 2014 to December 2014.

Of the 900 general practitioners, 141 consented to participate in data collection (90 general practitioners consented to participate in telephone interviews and 51 responded to a questionnaire). Of the general practitioners who consented to participate in telephone interviews, 13 were lost to follow-up (because of not responding, withdrawing consent due to time constraints or disagreement with our objectives), leaving 77 who completed the interview. They answered for the treatment of 268 patients (mean 2.2 patients per general practitioner) and diagnostic assessments for 339 patients (mean 2.7 patients per general practitioner). Of the 51 general practitioners who responded to the questionnaire, four were excluded (because the patients did not have depression according to the inclusion criteria), leaving 47 general practitioners who completed the questionnaire for one patient each (these patients did not receive a questionnaire). Thus, we collected data on the general practitioners’ management for 319 patients. We excluded 14 patients who did not meet our inclusion criteria, leaving treatment data from 305 patients for the analysis. In total, 124 of 900 general practitioners (response rate 14 %) participated in the data collection, 51 in the intervention group and 73 in the control group. Of 339 eligible patients, 134 responded to the questionnaire (response rate 40 %), 68 in the control and 66 in the intervention group. A significantly higher proportion of the participating general practitioners in the intervention group worked in rural practices. They also had an older patient group and fewer female elderly patients.

Characteristics of general practitioners, their practices and patients are summarised in Table 3.

**Table 3:** Characteristics of participating GPs, their practices and patients

Variables	Intervention	Control	*p*
General practitioners	*N* = 51	*N* = 73	
Clusters (municipalities)	26	28	
Female (%)	21 (41 %)	32 (43 %)	ns
Age (SD)	47.3 (11.39	49.4 (10.4)	ns
Year in practice (SD)	16.1 (11.5)	17.3 (10.9)	ns
Specialist in family medicine yes (%)	31 (61 %)	51 (70 %)	ns
Practices			
N^o^ of patients on list (SD)	1056 (352)	1084 (354)	ns
Large municipality/city (%)	9 (18 %)	43 (59 %)	<0.001
Many elderly on list (%)	39 (76 %)	42 (57 %)	0.03
Patients	*N* = 182	*N* = 203	
Female (%)	116 (64 %)	159 (78 %)	0.002
Age (SD)	73.9 (7.6)	75.1 (7.7)	ns
Mild depressive episode (F32.0)	21 (12 %)	35 (17 %)	ns
Moderate depressive episode (F32.1)	22 (12 %)	23 (11 %)	ns
Severe depressive episode (F32.2-3)	28 (15 %)	35 (17 %)	ns
Recurrent depression or dysthymia (F33.0-3, F34.1)	111 (61 %)	110 (55 %)	ns

*p* was calculated using chi-square test for dichotomous data and Student’s *t* test for continuous data. *ns* not significant on a 5 % level. Patient characteristics obtained from interview with GP

Representatives from 51 (64 %) of the 80 municipalities responded to the questionnaire. Forty-three municipalities (84 %) had developed a plan for psychiatry, but only four had included a care plan for elderly patients with depression. Five of the plans described collaboration between healthcare professionals within the municipality and between primary and specialist care for elderly patients with depression. Six municipalities provided specific referral forms for elderly patients with depression. However, most of the respondents commented that although the municipality did not possess a specific referral form for elderly patients with depression, they provided referral forms that applied to the whole population. Only three plans described collaboration between the healthcare system and voluntary organisations. Very few general practitioners were aware of a collaborative care plan in the municipality (three GPs in intervention and six GPs in the control group).

Amongst the 385 patients, 221 patients (58 %) suffered from recurrent depression according to the ICD-10 criteria. Of the remaining 164 patients with a first depressive episode, almost 40 % suffered from a severe episode.

### Primary outcomes

Mean adherence to the recommendations was 58 % (SD 20 %) in the intervention group and 53 % (SD 18 %) in the control group. The estimated difference in mean adherence from the univariate mixed model was 1.6 % (CI −6 to +9 %, *p* = 0.67). Multivariate linear regression analysis did not indicate that any of the potential effect modifiers could explain variations in effects (Table 4).

**Table 4:** Primary and secondary continuous outcomes reported by GPs and patients

	Intervention		Control		Estimated difference^a^ between intervention and control group (%) (lower CL (%), upper CL (%))
	*N*	Mean (%) (SD (%))	*N*	Mean (%) (SD (%))	
Primary outcome					
Mean adherence	51	58 (20)	73	53 (18)	2 (−11, 7)
Secondary outcome					
GP assessed CGI-I^b^	141	2.58 (1.04)	170	2.55 (1.04)	0.046 (−0.29, 0.38)
Patient assessed PGI^b^	55	2.20 (1.37)	69	2.10 (1.36)	0.18 (−0.47, 0.83)
HADS depression	60	6.55 (4.74)	70	6.83 (4.17)	−0.55 (−2.70, 1.60)
HADS anxiety	58	8.09 (4.86)	69	8.49 (4.11)	−0.075 (−2.01, 2.16)
HADS total	58	14.62 (9.01)	68	15.12 (7.64)	−0.38 (−4.27, 3.51)
Loneliness^c^	60	1.58 (1.11)	74	1.65 (1.00)	0.34 (−0.97, 0.30)
Sleeping problems improved^b^	31	2.32 (1.01)	43	2.05 (1.11)	0.57 (−0.06, 1.20)
Ability to cope with anxiety improved^b^	38	2.45 (1.16)	40	2.07 (1.39)	0.28 (−0.29, 0.91)
Problem solving^b^	53	2.79 (1.22)	68	2.54 (1.30)	0.28 (−0.34, 0.91)

^a^Estimated difference from final multivariate mixed model adjusting for size of municipality, proportion 80+ inhabitants in municipality, and potential effect modifiers with *p* value <0.3 in univariate analyses

^b^Mid-point 3, Likert-scale range 0-6, improvement indicated by lower than 3

^c^Better indicated by lower, range 0–3

*CGI-I* clinical global impression scale improvement, *CL* confidence level, *GP* general practitioner, *HADS* hospital and anxiety scale, *PGI* patient global impression

### Secondary outcomes

The clinical state of the patients after the intervention as assessed by general practitioners and patient responses is summarised in Table 4. There was little difference between the intervention and control groups for any of the secondary continuous outcomes, including depression, anxiety, loneliness, contact with voluntary organisations, physical activity, utilisation of self-help programmes and medication adherence.

There was little difference between the two groups for dichotomous outcomes (Table 5).

**Table 5:** Dichotomous secondary outcomes reported by patients

	Observed (raw data)				Estimated OR from final multivariate mixed model^a^		
	Intervention		Control		OR (control vs intervention)	Lower CL	Upper CL
	*n*	*N*	*n*	*N*			
Often/sometimes loneliness	34	60	46	74	1.81	0.43	7.66
Established contact voluntary organisations	11	59	14	73	3.01	0.97	9.30
More physically active	9	60	18	74	1.42	0.21	9.45
Self-help programme/literature	6	60	7	71	1.62	0.37	7.17
Adherence to antidepressant >0	28	60	26	74	1.02	0.26	4.05

^a^Estimated difference from final multivariate mixed model adjusting for size of municipality, proportion 80+ inhabitants in municipality, and potential effect modifiers with *p* value <0.3 in univariate analyses

However, there were wide confidence intervals for all of these outcomes.

### Post hoc analyses

We investigated the general practitioners’ adherence to the recommendations separately for mild, moderate, severe and recurrent depression. We also investigated adherence to individual recommendations. The difference in adherence varied from 15 % less adherence (for mild depression) to 7 % more adherence (for moderate depression) in the intervention group. However, the confidence intervals for all of these estimates were wide (Table 6). Adherence for the recommendation regarding improving social contact was high in both groups (75 % in the intervention group and 92 % in the control group), and this difference was significantly in favour of the control group in the univariate analysis. For the recommendation for “depression care manager” adherence was 16 % lower and for counselling adherence was 0.7 % higher. Confidence intervals were wide Additional file 1.

**Table 6:** Post hoc analysis of adherence to recommendations based on depression type and severity

	Intervention (observed in raw data)		Control (observed in raw data)		Estimated difference^a^ between intervention and control group (%) (lower CL (%), upper CL (%)) from final multivariate mixed model
	*N*	Mean (%) (SD (%))	*N*	Mean (%) (SD (%))	
Depressive episode					
Mild	15	49 (21)	26	55 (18)	−6 (−18, 6)
Moderate	16	49 (23)	17	45 (21)	3 (−18, 25)
Severe	18	49 (17)	22	51 (16)	−4 (−12, 20)
Recurrent depression/dysthymia	42	67 (26)	48	59 (24)	5(−7, 17)

^a^Estimated difference from final multivariate mixed model including four effect modifiers adjusting for size of municipality, proportion 80+ inhabitants in municipality, and potential effect modifiers with *p* value <0.3 in univariate analyses

A complete presentation of our analysis, with univariate and multivariate mixed models, including the four effect modifiers, is in the Additional file 1.

## Discussion

### Summary of findings

We have conducted a cluster randomised controlled trial, testing the effects of tailoring interventions to identified determinants of practice to improve adherence to evidence-based recommendations for managing depression in elderly patients. We were not able to recruit the required number of GPs for the data collection, and our study did not have sufficient statistical power to detect potential effects of the interventions. However, the upper limit of the 95 % CI for the primary outcome was 9 %, suggesting that the effect size on the primary outcome was moderate, at best.

### Strengths and limitations

The TICD project was an ambitious project that sought to address the effectiveness of tailoring implementation interventions to identified determinants of practice. It has contributed to the current knowledge base by adding results from five separate trials and process evaluations from five different countries, healthcare systems and disease groups [13]. Learning more about effective strategies to improve healthcare for patients with chronic diseases is important. This applies particularly to primary healthcare, because GPs are responsible for managing most patients with chronic diseases.

We used systematic approaches to identify determinants of adherence to the recommendations [36] and to tailor interventions to address prioritised determinants [23]. Tailored interventions are more effective than no interventions or dissemination of guidelines only, but further knowledge regarding how to use tailored implementation strategies is needed [12]. Cluster randomised trials are a robust study design for assessing the effects of implementation strategies [19]. This was a pragmatic trial [37], targeted at healthcare professionals, patients and their relatives. We randomised 80 municipalities and included all stakeholders in those municipalities without asking for consent to participate in the study, including all GPs in those municipalities and all the patients meeting our inclusion criteria.

The major limitation of our study is that we were not able to recruit a sufficient number of general practitioners to participate in the data collection within the time and resource limits that applied to our project. Thus, the study is underpowered and the results are inconclusive. Additionally, the low response rate resulted in samples that were not representative and might have biased the results, since it is possible that there were differences in general practitioners who participated in the data collection between the intervention and control groups. An alternative approach, seeking informed consent from the general practitioners prior to the intervention, might have resulted in a larger proportion of general practitioners participating in the data collection but would have made the study less pragmatic and limited the extent to which the results could confidently be applied to general practitioners who elected not to participate in the study.

The biggest challenge was to get in contact with the general practitioners due to them being guarded by the secretaries. When we had been able to talk to them, it was less difficult to get the general practitioner’s consent to participate in the data collection. In order to recruit the number of general practitioners, we estimated were necessary based on our sample size calculations, we had to include 20 % of the Norwegian municipalities and general practitioners. Despite using mailed letters, email messages, faxed invitations, and phone calls to practices, the response was disappointing. We did not have the resources to pay the general practitioners for the time spent in the telephone interview for the data collection. Getting in touch with more general practitioners (and getting past their secretaries) would have required more resources, including incentives for the general practitioners, and time than we had available to us. Prior to data collection, we reduced the required number of patients that we wanted to discuss with the general practitioners to four, anticipating that six patients, as described in the protocol, would be too exhaustive and time consuming. A further reduction to two, or even one, might possibly have increased the number of general practitioners who agreed to participate.

We used two different methods, interviews and questionnaires to obtain data from general practitioners regarding diagnosis and management of elderly patients with depression from their patient list. It is not possible to determine whether the two methods gave comparable results, due to the small sample size.

We did not collect baseline data due to the very short period of the TICD project that was planned for the intervention; collecting baseline data would have delayed the intervention substantially. Furthermore, we did not have the resources to collect such data.

Another important limitation of our study was that the study design, with a contemporaneous control group, prevented us from using the most common electronic dissemination resources, including the National Health Library, the Norwegian Directorate of Health and the Norwegian Electronic Medical Handbook. We received suggestions to use all of these during the design phase of our project [23]. Not putting the resources on a website that general practitioners frequently use may have limited the extent to which general practitioners used them. Another possible explanation for the modest use of the web resources is that new cases of elderly patients with depression are rare in clinical practice. Thus, general practitioners’ recall of this website or the perceived need to become familiar with it might have been low.

### Comparison with existing literature

There are few studies that measure adherence to depression guidelines, and they are not directly comparable to our study. A review found that general practitioners’ adherence to mental health clinical practice guidelines is low [38]. Fernandez and colleagues [39] found in a large epidemiological study based on interviews with 21,425 home-dwelling persons in six European countries, that “treatment adequacy” for depression as defined by the research group was particularly low (23 %) in the patient group that received management in “general medical care“(which included general practitioners and specialists other than psychiatrists and psychologists). Duhoux and colleagues [40] found that elderly patients (65+) received less guideline concordant management as compared with younger adults, regardless of which definition of concordance that were selected. Smolders and colleagues [9] combined information from a patient questionnaire that measured depression and anxiety symptoms and general practitioners’ performance as recorded in the electronic medical patient records that only 42 % of the depressed patients received management in accordance with evidence-based management of depression as defined by an expert panel. In our study, adherence to the recommendations tended to be higher than in previous studies that have reported adherence to depression guidelines, 58 and 52 % in intervention and control groups, respectively. The weak recruitment of general practitioners to participate in data collection might indicate that we were only able to collect data from general practitioners that were particularly interested in this patient group, both in the intervention and in the control group which may explain the relatively high adherence rate.

We are not aware of any systematic reviews or trials that address the implementation of clinical practice guidelines for elderly patients with depression. Baker and colleagues [12] reviewed 32 studies to assess the effectiveness of tailored implementation interventions. Fifteen studies were included in a meta-regression analysis, of which two addressed adherence to guidelines for depression in primary care. The meta-regression analysis demonstrated a modest effect on adherence. Data were insufficient to draw conclusions about healthcare outcomes and costs. In addition, the authors included 17 studies that were not eligible for the meta-regression analysis in the review. These studies supported the findings from the meta-regression analysis. Both studies that addressed depression management in primary care supported the general finding of the meta-regression analysis [41, 42].

Baker and colleagues [41] tailored an intervention based on psychological theories to improve implementation of a guideline for depression amongst general practitioners in a cluster randomised trial in UK. The research group identified obstacles to change from interviews with 64 general practitioners, grouped them within seven core domains and tailored an intervention to address these. This strategy yielded a higher proportion of general practitioners who assessed suicide risk and a larger proportion of patients who scored less than 11 on Beck’s depression inventory after 16 weeks, indicating remission. Callahan and colleagues [42] found that tailoring information for the management of elderly patients with depression yielded a higher proportion of patients receiving a depression diagnosis and an antidepressant.

In another systematic review, Williams and colleagues [7] included 28 randomised controlled multifaceted interventions to improve depression outcomes in primary care, including more than 1800 GPs and almost 11,000 patients. These interventions tested various multicomponent interventions involving a patient-directed component, and they reported a depression severity outcome. Although the overall effect of these interventions was in favour of the multifaceted interventions in the short term, the group concluded that patients with major depression and dysthymia (combined), extensive comorbidity or treatment resistant depression might require more intensive and lasting interventions.

Sinnema and colleagues [43] conducted a pragmatic cluster randomised controlled trial testing the effectiveness of a tailored implementation programme to improve recognition, diagnosis and treatment of anxiety and depression in primary care in the Netherlands. The intervention package was developed on the basis of interviews with all participating general practitioners in the intervention group, identifying a list of 84 barriers [44]. Various tailored interventions were implemented in two different formats; “peer group supervision” and “personalised telephone consultation”. They found that the intervention yielded a higher proportion of recognition of depression and anxiety and a higher number of consultations with the GP.

Richter-Sundberg and colleagues conducted a post-RCT qualitative study to identify barriers to the implementation of a clinical practice guideline for depression in Sweden. In spite of fees for performance and consent to participate collected prior to the implementation process, the project was not able to recruit the required number of patients to reach sufficient statistical power (after 18 months, only 30 patients were included) [45]. Based on the framework described by Grol and Wensing [46], the authors identified and sorted numerous barriers that hindered participation in the study. The excessive workload associated with the research design was one major barrier. Introducing new psychological therapies that challenged established professional role identity was another [45].

Although the findings of our study are inconclusive, the findings from the studies reported above indicate that tailored implementation strategies have a modest effect.

### Implications for clinicians and research

Our findings, which are inconclusive, do not have direct implications for clinicians. The challenges we encountered collecting data from general practitioners, which is the reason for our inconclusive findings, have implications for researchers. Although randomising general practitioners without their consent makes trials of implementation strategies more pragmatic, this creates problems if the data collection requires participation of the general practitioners. This might not be a problem if routinely collected data that is easily accessible can be used to measure outcomes. However, general practitioners do not routinely grade the severity of depression or the type of depression using diagnostic codes. Thus, collecting data on adherence to recommendations that apply to severity or type of depression requires an interview or completion of a questionnaire.

Beyond this, it is generally challenging to conduct both clinical and implementation research projects in primary care. A research network, with better infrastructure and incentives for general practitioners to participate, might facilitate recruitment of participants and data collection [47]. Alternatively, a fee for participating, either as part of national health authorities’ support of research in primary care or as part of the research project funding might have improved recruitment of participants for data collection [48].

Cluster randomised trials are considered a robust design for evaluating the effects of implementation strategies [49]. However, this study design prevented us from using the most common electronic dissemination resources as part of our intervention. A randomised design might not be appropriate if, as it did in this study, it places potentially important limitations on the implementation intervention. Interrupted time series studies might be a better design in these circumstances [50].

In the TICD project, we have conducted process evaluations to investigate reasons for the observed effects of our tailored implementation strategies, including the extent to which we were able to identify and address the most important determinants of practice, as described in the logic models that we created [51]. We will report the results from the process evaluation of the Norwegian trial in a separate report.

## Conclusions

It is logical to tailor implementation strategies to address determinants of practice. However, prior studies suggest that tailored interventions have a modest effect on professional practice, and it is uncertain whether those effects are any larger than the effects of interventions that were not tailored to address identified determinants of practice. The results of this study are consistent with those findings. Our tailored implementation strategy is unlikely to have had more than a modest effect, despite our having put substantial effort into using multiple methods to identify determinants of practice and to design a multifaceted implementation strategy to address those determinants. The TICD project is the first major research effort to compare different methods for identifying determinants of practice and designing interventions to address those. This study together with the other studies in the TICD project can inform future efforts to improve methods for tailoring implementation strategies to make them more effective.

Pragmatic trials of implementation strategies are needed to answer real world questions about how to improve the quality of care. A key message from this trial for implementation researchers is that access to outcome data is essential to their success. This trial included all general practitioners in 80 municipalities representing 20 % of the Norwegian population with close to 1000 general practitioners. Randomising jurisdictions or large numbers of practices without consent is a highly pragmatic approach to answering real world questions about how to improve the quality of care, provided access to outcome data is ensured, for example via routinely collected data. However, this approach proved to be fatally flawed in this trial, because collecting outcome data required active participation of general practitioners.

A second key message is that a randomised trial is not the best study design for answering a pragmatic question about how to improve practice when random allocation is not feasible. A major limitation of this trial was that we were not able to include what might have been important, effective components of our tailored implementation strategy, such as integrating our resources in widely used electronic information sources, because we could not randomly allocate these.

A third key message is that future research evaluating methods for tailoring implementation strategies should directly compare tailored implementation strategies that use different methods to tailor the interventions. It remains logical that implementation strategies should address important barriers to implementing evidence-based recommendations. However, little is known about how best to identify important barriers and how to select interventions to address identified barriers [12, 13]. The TICD project conducted some ground-breaking research comparing different methods for identifying determinants of practice [16] and for linking interventions to those determinants [14]. However, our trial was limited to comparisons of tailored strategies to no intervention. This research and process evaluations linked to our trial [51] can shed some light on why our tailored implementation strategy appeared to have, at best, modest effects. However, there is a need for explanatory trials designed to answer questions about how best to tailor implementation strategies, as well as pragmatic trials to answer real world questions about how to improve practice.

A key message for general practitioners and policymakers is that to answer important questions about how to improve practice, general practitioners need to have time, resources and structures, such as research networks, fees for participating in prioritised research or learning health systems [47, 52].

## Additional file

Additional file 1: A complete presentation of our analysis, with univariate and multivariate mixed models, including the four effect modifiers. (XLSX 20 kb)
